# The contextual separation of lateral white line patterns in chameleons

**DOI:** 10.1098/rsos.171235

**Published:** 2018-01-31

**Authors:** Tammy Keren-Rotem, Uri Roll, Amos Bouskila, Eli Geffen

**Affiliations:** 1 Department of Zoology, Tel Aviv University, Tel Aviv 69978, Israel; 2 Mitrani Department of Desert Ecology, The Jacob Blaustein Institutes for Desert Research, Ben-Gurion University of the Negev, Midreshet Ben-Gurion 84990, Israel; 3 Department of Life Sciences, Ben-Gurion University of the Negev, Beer-Sheba 84105, Israel

**Keywords:** badge, patch, ornament, visual signalling, social display, dual-function badge

## Abstract

While many animals display different colour patterns that signal different messages, some species use various tactics to separate between colour and pattern displays. The common chameleon (*Chamaeleo chamaeleon*) is capable of rapidly changing and separating among displays of colour patterns and ornaments. We used chameleons to study the contextual role of separation among colour and pattern displays. Specifically, we studied the predominant white badge, which is composed of multiple parts, during different seasons and in different social contexts. We hypothesized that the badge contains important information about the sender and, therefore, would be present during important social contexts. We carried out a series of trials to document the presence/absence of the badge and found that the badge is individually specific and reflects body size. We also revealed that the badge remained fixed throughout other body colour changes, but was replaced by other colour patterns during mating behaviour. During social encounters, additional dark patches delineating the badge appeared, presumably amplifying its signal. Thus, we suggest that the badge constitutes an important feature in intraspecific communication, and is possibly employed to display quality. However, the replacement of the badge by other displays during courtship suggests that during important social events like mating, chameleons transmit exclusive information that is not broadcast by the badge. Our findings demonstrate the importance of separation between colour patterns, and the alternative use of intraspecific colour patterns for specific social contexts in chameleons.

## 1. Introduction

Colour ornaments are a common way for signallers to transmit information regarding their traits to receivers. For example, an increase in the colour intensity of a patch might alter the attractiveness of the sender to the opposite sex (e.g. [1–5]). Colour ornaments could also signal competitive ability in males (e.g. [6]). Among animals that co-occur in the same area and are likely to interact repeatedly, colour ornament may enable individual recognition, with one individual (i.e. receiver) identifying another (i.e. sender) according to its distinctive phenotypic traits [7]. Individual recognition among conspecifics is advantageous, as it enables the occurrence of reciprocal altruism and assessment of fighting ability from previous encounters [8].

Studies have found that different colour patches on animals can signal different messages [9], which could be related to different habitats, environments [1,10] and distances [11,12]. In other cases, colour patches that are not relevant in specific situations are concealed and displayed only when required, such as when males seek to attract females (e.g. [13]), during begging by chicks (e.g. [14]), or when signalling to rivals (e.g. [15]). The question arises as to how animals trade off conflicting signalling motivations, and whether they need to compromise and give priority to one or more of the signals at the expense of other, conflicting signals.

**Figure 1.**
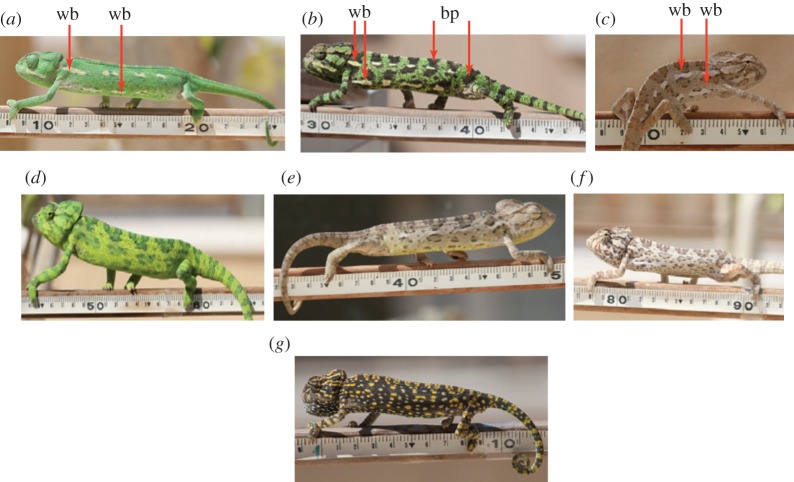
Individuals with a longitudinal white badge (wb) (*a,c*) and a white badge enhanced by contrasting dark patches (bp) (*b*). For comparison, mating-related colour patterns that lack the white badge (courting dominant males (*d*); courting sneaker males (*e--f*); non-receptive female (*g*)).

The common chameleon (*Chamaeleo chamaeleon*) is able to rapidly alter its body colour according to season, background matching and social signalling [16–18]. During the breeding season, males appear in green or brown colour patterns, which reflects whether they are dominant (figure 1*a*,*d*) or sneaker males (figure 1*c*,*e,f*). Chameleons thereby change colour patterns according to context, rather than concealing them. In addition to the interchangeable colour patterns displayed on their body, common chameleons regularly display an ornamentation of two distinct lateral white stripes [16], each composed of multiple parts of a series of white patches (hereafter referred to as the white badge; figure 1*a*–*c*); except for the desert subspecies, *C. c. musae*, which often has three such stripes [19]. However, observations revealed that this white badge is not on constant display and Cuadrado [16] reported one specific colour pattern, displayed by gravid females, in which the white badge was entirely absent. During the breeding season, non-receptive females do not present the white badge upon encountering a courting male, and change body pattern display to a specific conspicuous colour pattern that lacks this white badge [16]. Hence, chameleons have the ability to choose contextually between simultaneously displaying both one of the alternative colour patterns and the white badge, or displaying only one of the alternative colour patterns while eliminating the white badge. This ability of chameleons to separate between displays of colour patterns makes them a good model to investigate the contextual role of separation among colour pattern displays.

Based on previous observations on colour pattern interchange in a chameleon population we have been studying since 1999 [17,18], we hypothesized that (1) the white badge that chameleons regularly display simultaneously with interchangeable colour patterns could contain important information about the sender, and (2) if the white badge contains important information about the sender, it will not be eliminated during important social contexts (e.g. mate selection). In these social contexts, it will be displayed together with the contextual distinctive colour patterns.

## 2. Material and Methods

### 2.1. Study animals

The common chameleon is an arboreal lizard, highly adapted to life on plants. We used chameleons as a model to investigate the importance of temporal separation between signals because individuals alter their body colours and patterns according to context (e.g. sex, social status, social interactions, microhabitat and season; [17,18]). We conducted our study along the Maharal creek on the Mediterranean coast of Israel, at the foothills of Mt Carmel (32°38′N, 34°58′E). Fieldwork was carried out between May and November during 2008–2013. We collected chameleons from vegetation using a spotlight during the night, while they are asleep and their bodies are light in colour and reflective. Each chameleon was kept in a separate terrarium. All animals were measured, weighed, sexed and released back to their capture site the following day. We individually marked all animals just before their release by clipping off the tip of 1–3 nails using a fingernail cutter. This marking procedure took only a few seconds and the hand-held animals showed little resistance to it. The clipped nails regrew a rougher tip, which served to identify recaptures but did not affect the animals' ability to climb branches [20].

### 2.2. Recording presence/absence of the white badge

We carried out a series of trials to document the presence/absence of the white badge (i.e. two distinct lateral white stripes each composed of a single or a series of white patches, figure 1*a*–*c*) and the associated dark patches (figure 1*b*), using a total of 172 individuals. All trials were conducted in an arena comprising a 2.5 m high *Ficus benjamina* tree planted in a pot (29 cm high and 35 cm in diameter). This experimental set-up allowed individuals free movement in all directions on the tree. In our trials, we first placed each individual chameleon in the arena for 20 min. We recorded the specific colour pattern and the presence/absence of the white badge and dark patches of each individual by continuously recording colour changes throughout the entire trial interval. Next, we selected at random two individuals of either the same or different sex (male–male: *n* = 36, male–female: *n* = 60 and female–female: *n* = 25), and placed them in the arena together for 20 min. During this time, we recorded all the social interactions (i.e. agonistic interactions between males, courtship and mating events, responses to courting males by non-receptive females), and for each of the animals we noted the encounter-dependent colour patterns and the presence/absence of the white badge and dark patches. We lumped all the behaviours we observed into three categories. Mating denotes all behaviours associated with courtship or mating. Stay and Retreat refer to these actions during agonistic interactions between a pair of individuals. Trials were held during 2008–2013, both before and during the breeding season (i.e. we defined May–August as ‘before the breeding season', and September–October as ‘the breeding season'; [17,18]). The behaviour and colour changes of individuals in all trials were recorded using high-resolution images and video.

We examined the effects of season, sex, type of trial (single animal or social encounter) and type of encounter (male–male, male–female or female–female) on the presence/absence of the white badges and dark patches using logistic regression within the framework of the generalized estimating equation (GEE) approach. In this analysis, most animals were used in both trial types (once as a single animal and once in a social encounter), thus we set individual identity as a random effect in our models. GEE is an extension of generalized linear models for correlated data (i.e. a mixed model). The GEE approach results in estimates of model parameters that are robust regardless of correlation structure between observations (repeated measures within subjects). Wald χ^2^ statistics was used to test the significance of each effect in the model. During the breeding season, we also examined the effect of the mating behaviour context on the presence/absence of these colour patterns. All calculations were carried out in JMP (v. 12, SAS Institute Inc.) and SPSS (v. 23, IBM).

### 2.3. Measuring intraspecific variation in white badge pattern

A randomly selected subset of 22 individuals (11 males and 11 females), which we kept for 36 h before returning them to their natural habitat, were photographed twice, at a 24 h interval, to determine individual variation in white badge configuration. We individually placed each of these 22 chameleons on a 2 m long wooden stick, located horizontally 1 m above ground. The brown colour of the wooden stick simulated the colour of the branches that chameleons perch on in natural conditions. The animals often walked naturally along the stick, allowing us to record their colour patterns. In this position, the body is fully extended and the legs are extended away from the sides of the body. A white ruler aligned along the stick allowed us to accurately measure snout–vent length (SVL) of each individual [17]. We photographed both sides of the body using a Canon EOS D30 digital camera and macro lens (Canon 100–400 mm *f*/4.5–5.6 L IS USM). Photographs were saved in RAW format (7.5 Mb, 3504 × 2336 pixels). The camera was placed on a tripod 2 m from the focal animals. Photos were taken under natural sunlight, without a flash. Each photo included a colour standard in the form of the white ruler running along the horizontal stick. We standardized image colours by a ‘white standard' (approach resembles that of [21]), using the spectral reflectance of the white ruler [17] and the Photoshop software (v. 7, Adobe Systems, Inc.). Our approach also resembles that of Bergman & Beehner [22], in which photos were taken while recording presence/absence of the white badge and calibrated using the GretagMacBeth ColorChecker chart.

Next, we calculated the badge percentage overlap for all within and between individual pairwise combinations. To calculate overlap, we first manually outlined the border of the white badge in each image of the left side of the animals in order to minimize errors when comparing between pairs of photos. We then aligned each pair of photos to two fixed positions on the neck and cloaca of the chameleons. This provided a dorsal alignment of the badges, both minimizing differences due to varying body shapes or photo angles and negating potential body size-related differences. Overlap level between the white badges among all possible pairwise combinations (22 individuals, two photos per individual) were calculated using ArcGIS 10.1 (ESRI). We used the Behrmann equal area projection, calibrated on the first individual outlined, and based on this we matched all other individuals with a second order polynomial fit of additional control points used to align the two images. Overlap values were set into a 44 × 44 matrix.

To test for the presence of individual identity, we employed two statistical approaches. First, we used Ward's minimum variance method [23] to cluster all 44 outlined white badge patterns, based on the overlap matrix. Ward's approach is a popular agglomerative clustering method that uses sum-of-squares criterion to produce clusters of minimized within-group dispersion. Branch support was evaluated by the approximately unbiased *p*-values, which were calculated by multiscale bootstrap sampling using the R package *pvclust* [24]. Second, we calculated the ratio in mean overlap between and within individuals. To determine whether this ratio was significantly smaller than expected by random, we randomized the values in the overlap matrix in a manner similar to a Mantel's test, recalculated the ratio between the overlap means, and repeated the process 1000 times. To determine the *p*-value, we calculated the percentile of the observed overlap ratio in the distribution of randomized overlap values.

## 3. Results

### 3.1. Display frequency of the white badge and the dark patches

**Figure 2.**
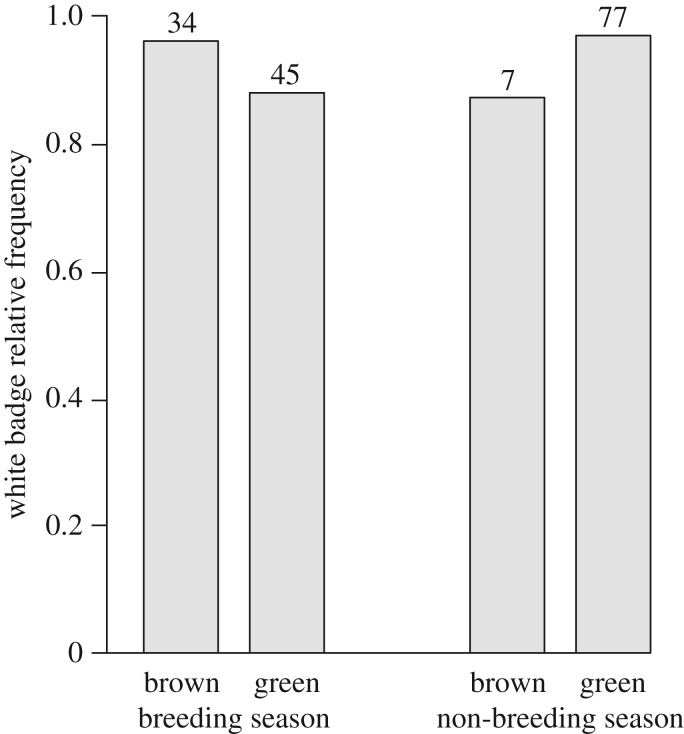
White badge relative frequency during the breeding and non-breeding seasons in green and brown individuals. Sample size is denoted above bars.

In this study, we documented the presence of a distinct white badge (figure 1*a*–*c*) and the dark patches that emphasized it on the chameleon body (figure 1*b*). Our analysis showed that overall the white badge was equally observed on both the animals that were placed alone in the arena and on those that participate in a social encounter (relative frequency of 0.96 and 0.94 for solitary animals and during social encounters, respectively; figure 2), but there were seasonal and sexual differences (table 1). By contrast, the presence of dark patches was significantly dependent on the social state (relative frequency of 0.16 and 0.32 for solitary animals and during social encounters, respectively; table 1). Owing to these frequency differences, we analysed the data separately for solitary individuals and for those that were in a social encounter (table 2)

**Table 1.** The effect of sex, season (before and during the breeding season) and social state (solitary individual and social encounter) on the presence of the white badge and dark patches. Individual identity was assigned as random effect in both GEE models. *n* = 416.

effect	estimate	Wald $\chi_{1}^{2}$	*p*
*white badge*			
sex	1.419	6.9	0.008
season	−1.234	4.4	0.036
social state	−0.525	2.5	0.111
*dark patches*			
sex	0.328	1.7	0.195
season	−0.641	5.9	0.015
social state	0.865	12.3	0.000

**Table 2.** The effect of sex, season (before and during the breeding season), and colour pattern (green or brown) on the presence of the white badge and dark patches in solitary individuals and during social encounter. Individual identity was assigned as random effect in both GEE models. *n* = 416.

	solitary individuals			during encounter		
effect	estimate	Wald $\chi_{1}^{2}$	*p*	estimate	Wald $\chi_{1}^{2}$	*p*
*white badge*						
sex	1.437	2.6	0.105	1.627	7.3	0.007
season	−1.606	2.9	0.090	−1.503	5.7	0.017
body colour	0.949	0.8	0.365	0.891	2.6	0.103
*dark patches*						
sex	−0.915	3.9	0.047	0.545	3.2	0.074
season	1.211	7.5	0.006	−0.731	5.4	0.021
body colour	−3.161	8.0	0.005	−1.664	15.1	<0.001

The frequency of white badge display in animals that were placed alone in the arena was not significantly different between sexes, before and during the breeding season, and on green and brown body colour (table 2; figure 2). However, white badge display during social encounter was greater in females (relative frequency of 0.98 and 0.90 for females and males, respectively) and before the breeding season (relative frequency of 0.98 and 0.90 before and during the breeding season, respectively). The display of the white badge was most common in the female–female encounters before and during the breeding season, but we did not detect significant differences in the white badge frequencies among the sex combinations (male–male, male–female and female–female; $\chi_{2}^{2}$ = 3.4, *p *= 0.182 and $\chi_{2}^{2}$ = 1.7, *p *= 0.416 for before and during the breeding season, respectively). No frequency difference was detected between green and brown body colour (relative frequency of 0.93 and 0.97 for green and brown body colour, respectively; table 2).

In the solitary animals, the dark patches were more frequent in males (relative frequency of 0.04 and 0.08 for females and males, respectively), during the breeding season (relative frequency of 0.03 and 0.10 before and during the breeding season, respectively), and in the green colour individuals (relative frequency of 0.22 and 0.01 for green and brown body colour, respectively; table 2). During social encounters, we did not detect a difference in the frequency of the dark patches between the sexes (relative frequency of 0.29 and 0.19 for females and males, respectively), but observed a higher frequency before the breeding season (relative frequency of 0.31 and 0.18 before and during the breeding season, respectively) and in the green colour individuals (relative frequency of 0.42 and 0.12 for green and brown body colour, respectively; table 2). The frequency of dark patches was not different between sex combinations in encounters before ($\chi_{2}^{2}$ = 4.2, *p *= 0.122) and during the breeding season ($\chi_{2}^{2}$ = 4.1, *p *= 0.126).

**Figure 3.**
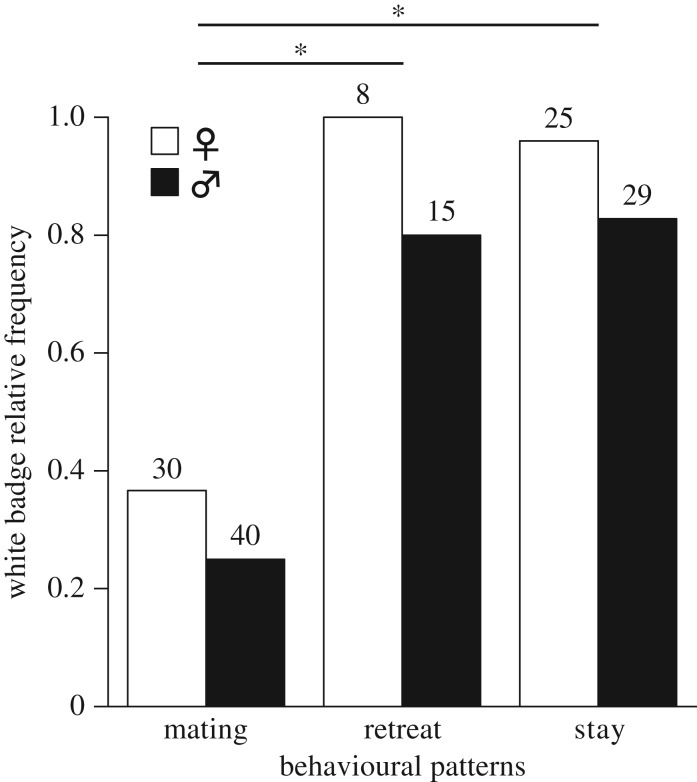
White badge relative frequency during the breeding season in males and females in different behavioural contexts. Mating denotes all behaviours associated with courtship or mating. Stay and Retreat refer to these actions during agonistic interactions between a pair of individuals. Asterisk denotes *p *< 0.0001.

In the breeding season, we observed diverse mating and non-mating behaviours during encounters between individuals. These behaviours included agonistic interactions between males, courtship and mating events and responses to courting males by non-receptive females. Specific colour patterns were associated with courtship in males (figure 1*d*–*f*), and male rejection behaviour in non-receptive females (figure 1*g*). In all behaviour patterns, females displayed the white badge significantly more frequently than males (logistic regression, $\chi_{1}^{2}$ = 4.5, *p *= 0.034; figure 3). During mating-related behaviours, the probability of displaying the white badge by individuals (both males and females) was only 0.3 (figure 3). In contrast, the probability of displaying the white badge by both males and females during non-mating behaviour was significantly higher (logistic regression, $\chi_{1}^{2}$ = 41.7, *p* < 0.0001); 0.87 during the retreat behaviour and 0.91 during the stay (i.e. remaining in the experimental set-up) behaviour (figure 3).

### 3.2. Individual-specific white badge

**Figure 4.**
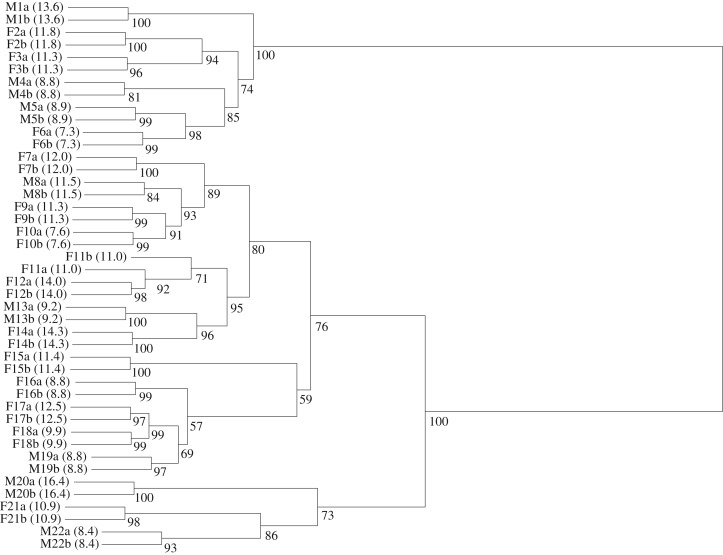
Ward's hierarchical cluster analysis of individuals based on the white badge overlap. The first letter of the individual identity denotes sex (F for female and M for male) and the animal size (SVL) is in parentheses. The letters ‘a' and ‘b' at the end of the individual identity denote repeated samples from the same individual. The approximately unbiased *p*-values are shown below nodes.

The white badge was individually specific. Ward's minimum variance hierarchical method correctly clustered all the images of the white badge to individuals, except for one case (F11, figure 4). Most of the bootstrap approximately unbiased *p*-values (19 out of 22 cases) for within-individual nodes are above 0.9, and two additional nodes are above 0.8 (figure 4). These results imply that the within-individual clustering is statistically robust. Further, the observed ratio in mean overlap between and within individuals was 0.299, indicating that the mean overlap within individuals was three times greater than between individuals. This overlap ratio was significantly smaller than that expected randomly (*p* < 0.0001).

The total length of the white badge correlated with snout–vent length (SVL; left side, upper white stripe: *r*^2 ^= 0.699, *F*_1,69 _= 11.9, *p* < 0.0001; left side, lower white stripe: *r*^2 ^= 0.520, *F*_1,69 _= 7.7, *p* < 0.0001; right side, upper white stripe: *r*^2 ^= 0.632, *F*_1,69 _= 10.4, *p* < 0.0001; right side, lower white stripe: *r*^2 ^= 0.607, *F*_1,69 _= 9.6, *p* < 0.0001). We did not detect an interaction between sex and SVL in any of the above cases (*p* ≥ 0.074). The number of white segments that comprised the white badge did not correlate with SVL (on either left or right side of the body; *r*^2^ ranged 0.013–0.033, *p* ≥ 0.248), except for the number in the lower right stripe, which significantly correlated with SVL (*p* = 0.045) but accounted for only 7.7% of the variation. In addition, the white badge shape did not cluster according to body size or sex (figure 4). These findings imply that while the size of the white badge is proportional to the total body length (i.e. SVL), its shape, based on percentage overlap between individuals, conveys no information on the size or sex of the animal.

## 4. Discussion

### 4.1. Potential information retained in the white badge

#### 4.1.1. Individual recognition

Our findings reveal that male and female chameleons consistently display a white badge, which generally remains even when shifting between body colours and patterns. We also found that the white badge is stable in shape within individuals but varies between individuals. Such a type of badge could potentially be used to enable individual recognition (though we have not tested this specifically in this study). Furthermore, Gherardi *et al*. [25], suggest that chameleons, being non-social animals, do not gain any meaningful advantage from individual recognition capability. Nevertheless, individual recognition, is very widespread in the animal kingdom and has been documented in a variety of taxa, including insects [7,26], fish [27], reptiles [28], birds [29,30] and mammals [31,32]. The identification of particular individuals could be advantageous only when individuals repeatedly meet and interact with the same conspecifics [25]. Thus, we cannot conclude that the chameleon's white badge is indeed used for individual recognition, but only suggest that it bears the information necessary for such a function.

#### 4.1.2. Individual quality

Chameleons display the white badge almost continuously both before and during the breeding season, and also when individuals are alone as well as during social encounters. Our findings also indicate that the white badge is proportional to the total body length, and thus its length could reflect or even emphasize (i.e. amplifier signal) body size [33,34]. Such a type of badge could also potentially be used for signalling individual quality. Studies on lizard species have indicated that body size is used to assess an opponent's fighting ability [35] and dominance [36]. Taylor *et al*. [37] suggested that a characteristic pattern might serve as a standard enabling comparison among different individuals. Another indication of the importance of the white badge in communication was the more frequent appearance of dark patches delineating it during encounters (figure 1*b*). Hasson [33] argued that when a particular colour patch or pattern amplifies a message, it might also improve the perception and processing of another proximate cue or signal and, consequently, improve the transmission of the message. For example, the dark colour around the orange ornaments in guppies serves as a cue amplifier [38]. Accordingly, we suggest that the dark patches displayed by chameleons could amplify the white badge.

Andersson [39] argued that individuals might employ badges that reveal the overall quality of the signaller in order to attract mates (i.e. physical condition, parental care abilities, territory quality, age, experience, good genes and freedom from disease). For example, the medial line and vertical bar displays used during contests between cichlid fish are thought to visually amplify both the size and condition of individuals [40]. Similarly, we suggest that the white badge could have an important role in communication among common chameleons and, as we hypothesized, could constitute a badge transmitting information on the individual's quality.

We propose that the white badge could even indicate both the identity and the quality of an individual chameleon, hence possessing a dual function. Studies on dual-function ornaments show that they function both in male–male agonistic encounters and in female choice [41,42]. However, our propositions require further experimental work to confirm that chameleons respond differently to known neighbours and strangers, and that indeed the white badge size and contrast is reflecting other quality traits than size alone.

### 4.2. Contextual appearance of colour patterns

Colour patches for sexual selection communication are often conspicuous because of their role in mate choice and sexual competition [39]. However, they also increase the risk of detection by predators (e.g. [43–46]). In order to both reduce detection by predators and increase detection by conspecifics, a wide range of animals conceal their conspicuous body colours and expose them intentionally only during relevant social communication (e.g. [47–52]). Unlike most animals, chameleons do not need to conceal the conspicuous colour patterns on their body parts because they have the ability to change colours and patterns much like an electronic billboard that alternates momentarily between advertisements, changing the entire colour and pattern. In the present study, we have shown experimentally that chameleons, which otherwise displayed the white badge almost constantly, replaced it by other patterns in certain social contexts, and specifically during mating behaviours. These findings contradicted our initial hypothesis that the white badge, if containing important information about the sender, would be displayed during key events like mating and would be displayed simultaneously with contextually distinctive colour patterns. This raises the question—what is the possible role of the white badge in chameleons?

A recent comparative study on many chameleon species suggests that the lateral lines serve as a secondary sexual signal [53]. In their study, Resetarits & Raxworthy [53] found a positive correlation between the presence of the ventral or lateral lines and arboreal habits. They also showed in a series of behavioural trials on *Chamaeleo viridis* that the lateral lines were hidden from the predator and were observed only on males. Lateral displays are positively associated with fighting ability in other male chameleon species (*Bradypodion pumilum*; [54]). Taken together, the findings by Resetarits & Raxworthy [53] suggest that lateral lines function as a secondary sexual signal in males and may not have an antipredator function, and that arboreal habitats increase the signal efficacy of these lateral lines to conspecifics. These findings, however, contradict our own observations on the common chameleon which demonstrated that during courtship events lateral lines are not presented. Therefore, the lateral white lines in the common chameleon are unlikely to play a role as a secondary sexual signal.

We have explained our findings by the multitasking hypothesis [55], which suggests that the information transfer in one colour pattern is constrained by the presence of another colour pattern, thus using both simultaneously is difficult (i.e. a tight negative correlation). Consequently, a multitasking ability might be advantageous in assessing a signaller's quality but not when signalling mating intentions or female receptivity. We did observe both the white badge and various mating patterns (figure 1*d*–*f*) together on the same individual but at low frequency (figure 3). Under this hypothesis, the white badge transmits general information regarding an individual's quality, but may not be sufficiently clear to transmit mating intentions. Thus, during courtship and mating, the white badge is replaced by a specific pattern, which could transmit a definite message of male or female intentions and is independent in appearance and probably also in information content.

Future studies with painted individuals and additional field trials or controlled experiments in a semi-natural setting may be required in order to fully resolve the function of the white lateral lines in chameleons. Nevertheless, our findings demonstrate an alternative use of intraspecific colour patterns for specific social contexts. Chameleons alternate between different displays rather than concealing or exposing the same display via other body parts. We have demonstrated a clear separation between colour patterns used for communication among conspecifics, which suggests that the separation between intraspecific colour patterns constitutes a mechanism for enhancement clarity during important social interactions.

## Supplementary Material

White badge overlap with and between pairwise combinations

## Data accessibility

Raw data are available in the electronic supplementary material.
